# A Manual of Surgery—General and Operative

**Published:** 1895-02

**Authors:** 


### Book Reviews.

---

A Manual of Modern Surgery—General and Operative. By John Chalmers Dacosta, M. D., Demonstrator of Surgery Jefferson Medical College, Philadelphia; Chief Assistant Surgeon Jefferson Medical College Hospital, Etc. With 188 Illustrations in the Text, and 13 Full-page Plates in Colors and Tints, Aggregating 276 Separate Figures. Philadelphia: W. B. Saunders, 925 Walnut Street. 1894.

This manual is a part of "Saunders' New Aid Series" of books for students, and meets well the requirements of a text-book. The portion devoted to fractures and to bandaging is worthy of special commendation. The features embodied in this book may be mentioned as follows:

1. Comprehensiveness.
2. Comprehensibleness.
3. Conciseness.
4. Conservativeness.

Gross and the founders of surgery are quoted and expounded rather than every aspirant for surgical honors. We regard a book of this nature as a great possession for a student, weary of work and worry, and anxious to learn the landmarks of surgery. J. McF. G., Jr.

